# Survival outcomes and clinical characteristics of brain metastases from prostate cancer: A single-center analysis

**DOI:** 10.1093/noajnl/vdaf063

**Published:** 2025-03-22

**Authors:** Kurl Jamora, Adeodatus Vito Nicanor, Ayah Erjan, Marc Vincent Barcelona, Dana Keilty, Michael Yan, Aristotelis Kalyvas, Marc Bernstein, Paul Kongkham, Gelareh Zadeh, Eshetu Atenafu, Srinivas Raman, Alejandro Berlin, Charles Catton, Peter Chung, Barbara-Ann Millar, Normand Laperriere, Tatiana Conrad, David Shultz, Enrique Gutierrez-Valencia

**Affiliations:** Radiation Medicine Program, Princess Margaret Cancer Centre, University Health Network, Toronto, Ontario, Canada; Department of Radiation Oncology, University of Toronto, Toronto, Ontario, Canada; Radiation Medicine Program, Princess Margaret Cancer Centre, University Health Network, Toronto, Ontario, Canada; Department of Radiation Oncology, University of Toronto, Toronto, Ontario, Canada; Radiation Medicine Program, Princess Margaret Cancer Centre, University Health Network, Toronto, Ontario, Canada; Department of Radiation Oncology, University of Toronto, Toronto, Ontario, Canada; Radiation Medicine Program, Princess Margaret Cancer Centre, University Health Network, Toronto, Ontario, Canada; Department of Radiation Oncology, University of Toronto, Toronto, Ontario, Canada; Radiation Medicine Program, Princess Margaret Cancer Centre, University Health Network, Toronto, Ontario, Canada; Department of Radiation Oncology, University of Toronto, Toronto, Ontario, Canada; Radiation Medicine Program, Princess Margaret Cancer Centre, University Health Network, Toronto, Ontario, Canada; Department of Radiation Oncology, University of Toronto, Toronto, Ontario, Canada; Division of Neurosurgery, Toronto Western Hospital—University of Toronto, Toronto, Ontario, Canada; Division of Neurosurgery, Toronto Western Hospital—University of Toronto, Toronto, Ontario, Canada; Division of Neurosurgery, Toronto Western Hospital—University of Toronto, Toronto, Ontario, Canada; Division of Neurosurgery, Toronto Western Hospital—University of Toronto, Toronto, Ontario, Canada; Department of Biostatistics, Princess Margaret Cancer Centre, University Health Network, Toronto, Ontario, Canada; Radiation Medicine Program, Princess Margaret Cancer Centre, University Health Network, Toronto, Ontario, Canada; Department of Radiation Oncology, University of Toronto, Toronto, Ontario, Canada; Radiation Medicine Program, Princess Margaret Cancer Centre, University Health Network, Toronto, Ontario, Canada; Department of Radiation Oncology, University of Toronto, Toronto, Ontario, Canada; Radiation Medicine Program, Princess Margaret Cancer Centre, University Health Network, Toronto, Ontario, Canada; Department of Radiation Oncology, University of Toronto, Toronto, Ontario, Canada; Radiation Medicine Program, Princess Margaret Cancer Centre, University Health Network, Toronto, Ontario, Canada; Department of Radiation Oncology, University of Toronto, Toronto, Ontario, Canada; Radiation Medicine Program, Princess Margaret Cancer Centre, University Health Network, Toronto, Ontario, Canada; Department of Radiation Oncology, University of Toronto, Toronto, Ontario, Canada; Radiation Medicine Program, Princess Margaret Cancer Centre, University Health Network, Toronto, Ontario, Canada; Department of Radiation Oncology, University of Toronto, Toronto, Ontario, Canada; Radiation Medicine Program, Princess Margaret Cancer Centre, University Health Network, Toronto, Ontario, Canada; Department of Radiation Oncology, University of Toronto, Toronto, Ontario, Canada; Radiation Medicine Program, Princess Margaret Cancer Centre, University Health Network, Toronto, Ontario, Canada; Department of Radiation Oncology, University of Toronto, Toronto, Ontario, Canada; Radiation Medicine Program, Princess Margaret Cancer Centre, University Health Network, Toronto, Ontario, Canada; Department of Radiation Oncology, University of Toronto, Toronto, Ontario, Canada

**Keywords:** brain metastasis, overall survival, prostate cancer

## Abstract

**Background:**

Brain metastases (BrM) from prostate cancer (PC) are rare. This study sought to evaluate their prevalence, clinical features, treatment modalities, and survival outcomes.

**Methods:**

From a database of BrM patients, we analyzed 28 cases of prostate cancer treated at our center between 2008 and 2023.

**Results:**

BrM from PC comprised 0.7% of cases. The majority of patients had high-risk features at PC diagnosis: median prostate-specific antigen (PSA) at diagnosis was 65.5 ng/ml (range: 3.9–784.7 ng/ml), 82% were Gleason grade group ≥ 4, and 68% had perineural invasion (PNI). At BrM diagnosis, 79% were castrate-resistant. Most patients had concurrent metastases, including bone (94%), lymph nodes (63%), or lung (6%). Fifty percent presented with a single brain lesion, and the median Graded Prognostic Assessment (GPA) score was 1.5 (range: 0.5–2.5). Patients commonly had radiographic brain edema (57%) and neurological symptoms (54%), whereas only 7% had seizures. Median overall survival (OS) was 9.4 months (95% CI: 4.8–14.8 months) after BrM diagnosis. An upward trend in OS was observed with higher GPA (*P* = .07). Treatment modalities, including surgery with adjuvant radiation, stereotactic radiosurgery, and whole brain radiotherapy, showed no significant difference in median OS (9.4, 10.1, and 11.0 months respectively, *P* = .79). OS did not significantly differ between patients with a single versus multiple BrM or patients with castrate-sensitive versus castrate-resistant PC.

**Conclusion:**

BrMs from prostate cancer are rare and predominantly occur in patients with advanced, castrate-resistant disease, often accompanied by other metastases. This analysis enhances our understanding of the disease trajectory and informs treatment discussions.

**Key Points**

- Brain metastases from prostate cancer are rare, with an incidence of 0.7% in our study.
- They more commonly occur in patients with advanced, castrate-resistant disease.
- Median OS was 9.4 months after BrM diagnosis and did not differ by treatment received.

**Importance of the Study**

The rising incidence of brain metastasis can be attributed to improved overall survival in patients with various carcinomas. However, most research has focused on lung, melanoma, and breast cancers. In contrast, brain metastases from prostate cancer are relatively uncommon, with incidence rates reported between 0.16% and 8.06%. Our study reveals an incidence of 0.7%, primarily in patients with advanced, castrate-resistant disease, often alongside other metastatic sites. This manuscript offers valuable insights into the limited existing literature regarding the incidence, clinical features, survival outcomes, and treatment options for brain metastases stemming from prostate cancer. Furthermore, it highlights the potential use of the Graded Prognostic Assessment (GPA) as a tool for estimating overall survival.

Brain metastases (BrM) are commonly observed and extensively studied in lung, melanoma, and breast cancers.^1–3^ Conversely, prostate cancer (PC) rarely metastasizes to the brain. Limited retrospective reviews and institutional reports have documented its incidence between 0.16% and 8.06%.^4^ Advancements in brain imaging and more effective treatment options for PC and BrM may alter their incidence rates and clinical outcomes, underscoring the necessity for further research on this uncommon phenomenon. This study aims to assess the prevalence, clinical characteristics, treatment options, and survival outcomes of BrM from PC.

## Methods

### Patients

From a prospectively maintained database of BrM patients at Princess Margaret Cancer Centre, covering treatments from January 2008 to December 2023, we identified patients with histologically proven PC and excluded those with second primary cancers or parenchymal invasion from adjacent skull-based disease. We determined patient demographics, clinical details on PC and BrM diagnosis, and clinical outcomes.

### Statistical Analysis

Categorical variables, such as perineural invasion, distant metastasis at diagnosis, bone metastasis, lung metastasis, lymph node metastasis, ECOG at BrM diagnosis, neurological symptoms (eg, headache, nausea, vomiting, sensorimotor deficits, or seizures caused by edema or intracranial pressure changes), brain edema, seizures, systemic therapy at BrM diagnosis, type of BrM, location of BrM, and type of BrM treatment, were presented as counts and percentages. BrM types were categorized as pure parenchymal metastases (confined to the brain parenchyma without involvement of the pachymeningeal dura), pure dural metastases (originating from the pachymeningeal dura without bony skull involvement), and both (involving both intraparenchymal and dural sites). Continuous variables such as age at diagnosis, total GPA score, as well as follow-up were presented as medians with ranges.

The main outcome variable of interest includes time to death (OS). Time to death was calculated in months from the BrM diagnosis (Date of Brain Mets) to the date of death or to the date of the last follow-up, whichever comes first. Overall survival rates were calculated using the Kaplan–Meier product-limit method and the log-rank test was used to assess the impact of covariates of interest on OS. The Cox proportional hazards model was used to obtain HR rates with 95% CI. All *P*-values will be 2-sided and for the statistical analyses, *P* < .05 will be considered to indicate a significantly different result. Statistical analyses will be performed using SAS version 9.4 of the SAS system for Windows (Copyright © 2002-2012 SAS Institute, Inc., Cary, NC. USA).

## Results

### Proportion of Brain Metastases from Prostate Cancer

**Figure 1.**
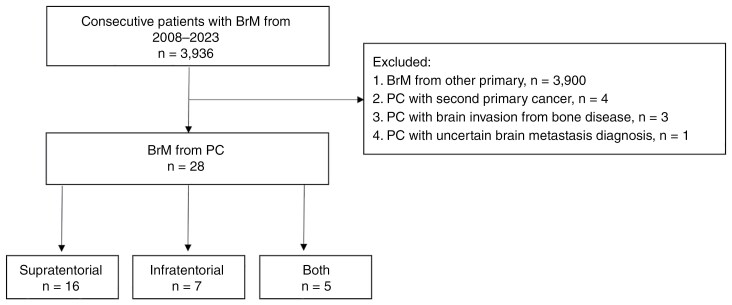
Flow diagram summarizing patient selection. BrM: brain metastasis; PC: prostate cancer.

Of the 3,936 patients with BrM seen at our institution between January 2008 and December 2023, 36 had PC. Of these patients, 4 had a second primary cancer, 3 had skull-based metastasis with adjacent parenchymal invasion, and 1 had an uncertain diagnosis of BrM, which could potentially be a meningioma (Figure 1). Following multidisciplinary discussion, consensus based on imaging suggested meningioma for the latter patient, leading to a decision for observation. The proportion of BrM originating from PC is therefore 0.7% (28 out of 3936) with 11 (39.3%) of these being pathologically confirmed. More than half (15, 54%) presented with neurologic symptoms, while the remainder were incidentally discovered on imaging.

### Patient and Tumor Characteristics

**Figure 2.**
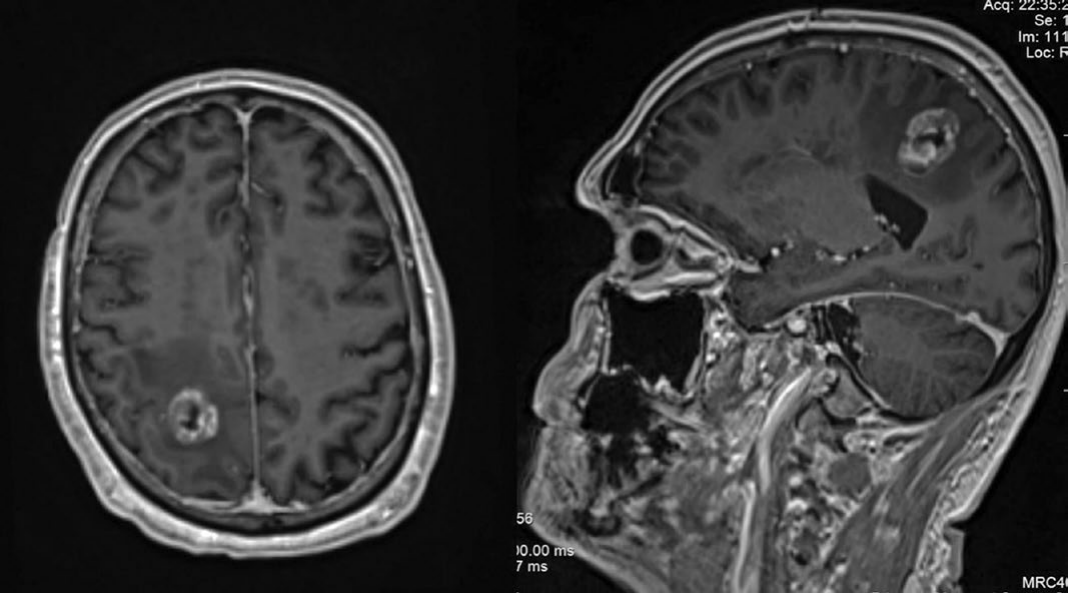
Intraparenchymal brain metastasis. Axial and sagittal T1-weighted MR images showing a heterogeneous enhancing metastasis in the right posterior semiovale, accompanied by surrounding vasogenic edema.

**Figure 3.**
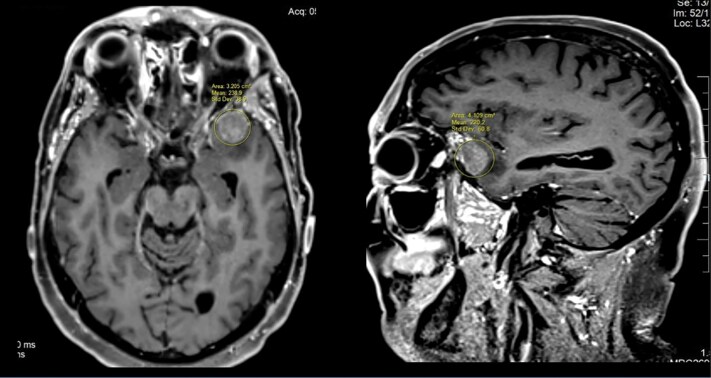
Purely dural-based metastasis. Axial and sagittal T1-weighted MR images showing an enhancing dural-based mass in the anterior left middle cranial fossa. The lesion does not involve the overlying skull.

Patient and disease characteristics of the 28 patients included in the study are listed in Table 1. The median age at BrM diagnosis was 63 years (range: 51–81 years). All patients had adenocarcinoma histology. At the time of PC diagnosis, median prostate-specific antigen (PSA) was 65.5 ng/ml (range: 3.9–784.7 ng/ml). All had a Gleason score of ≥ 7, and a majority of 23 (82%) had a Gleason score of ≥ 8. The majority of patients (17, 68%) had perineural invasion, and 15 (54%) presented with *de novo* metastatic disease. At the time of BrM diagnosis, 22 patients (79%) were castrate-resistant. BrM diagnosis occurred at a median of 47.8 months (range: 0.1–241 months) from PC diagnosis. Most had concurrent metastases, including bone 15 (94%), lymph nodes 10 (63%), or lung 1 (6%), and 23 (82%), were receiving systemic therapy. Most of the patients (16, 64%) had an ECOG performance status of 0–1. Fifty percent of patients (14) presented with a single brain lesion, and the median Graded Prognostic Assessment (GPA) score was 1.5 (range: 0.5–2.5). Patients had either pure intraparenchymal metastasis (*n* = 12, 42.9%, see Figure 2), pure dural metastasis (*n* = 15, 53.6%, see Figure 3), or both (*n* = 1, 3.6). Metastases were supratentorial only in 16 (57%) patients, infratentorial only in 7 (25%), and both supratentorial and infratentorial in 5 (18%). Patients commonly had neurological symptoms (15, 54%) and brain edema on imaging (16, 57%), whereas only 2 (7%) had seizures.

**Table 1.** Clinical and Treatment Characteristics of Prostate Cancer Patients with Brain Metastases BrM: brain metastasis; CSPC: castrate-sensitive prostate cancer; CRPC: castrate-resistant prostate cancer; ECOG: Eastern Cooperative Oncology Group; GPA: Graded Prognostic Assessment; PC: prostate cancer; PSA: prostate-specific antigen; SRS: stereotactic radiosurgery; VMAT: volumetric modulated arc therapy; WBRT: whole brain radiotherapy

	*N* = 28
Age at diagnosis	
Mean (SD)	63.6 (8.5)
Median (Range)	62.6 (51.0, 81.4)
PSA at diagnosis	
Mean (SD)	130.1 (195.5)
Median (Range)	65.5 (3.9, 784.7)
Gleason score, *n* (%)	
7	5 (17.9%)
8	12 (42.9%)
9	10 (35.7%)
10	1 (3.6%)
Perineural invasion, *n* (%)	
Yes	17 (68.0%)
No	8 (32.0%)
Missing	3
Distant metastasis at diagnosis, *n* (%)	
Yes	15 (53.6%)
No	13 (46.4%)
Extracranial disease, *n* (%)	
Absent	2 (7.1%)
Present	26 (92.9%)
Bone metastasis, *n* (%)	
Yes	15 (93.8%)
No	1 (6.3%)
Missing	12
Lung metastasis, *n* (%)	
Yes	1 (6.3%)
No	15 (93.8%)
Missing	12
Lymph node metastasis, *n* (%)	
Yes	10 (62.5%)
No	6 (37.5%)
Missing	12
CSPC or CRPC, *n* (%)	
CSPC	6 (21.4%)
CRPC	22 (78.6%)
Number of BrM at diagnosis, *n* (%)	
1	14 (50.0%)
2	4 (14.3%)
3	2 (7.1%)
4	4 (14.3%)
5	1 (3.6%)
6	1 (3.6%)
10+	2 (7.1%)
Months from PC diagnosis to BrM	
Mean (SD)	77.8 (73.4)
Median (Range)	47.8 (0.1, 240.9)
ECOG at BrM diagnosis	
0	3 (10.7%)
1	15 (53.6%)
2	6 (21.4%)
3	4 (14.3%)
Total GPA score	
Mean (SD)	1.4 (0.6)
Median (range)	1.5 (0.5, 2.5)
Neurological symptoms, *n* (%)	
No	13 (46.4%)
Yes	15 (53.6%)
Brain edema, *n* (%)	
No	12 (42.9%)
Yes	16 (57.1%)
Seizures, *n* (%)	
No	26 (92.9%)
Yes	2 (7.1%)
Systemic therapy at BrM diagnosis, *n* (%)	
Yes	23 (82.1%)
No	5 (17.9%)
Type of BrM, *n* (%)	
Pure intraparenchymal BrM	12 (42.9%)
Pure dural BrM	15 (53.6%)
Both intraparenchymal and dural BrM	1 (3.6%)
Location of BrM, *n* (%)	
Supratentorial only	16 (57.1%)
Infratentorial only	7 (25.0%)
Both supratentorial and infratentorial	5 (17.9%)
Type of BrM treatment, *n* (%)	
SRS	7 (25.0%)
WBRT	6 (21.4%)
Posterior fossa radiotherapy	2 (7.1%)
Surgery + SRS/VMAT	7 (25.0%)
Surgery + WBRT	4 (14.3%)
Palliative care	2 (7.1%)

### Survival

All 28 patients were analyzed for treatment and survival outcomes. Patients received the following treatments: stereotactic radiosurgery (SRS) (*n* = 7), whole brain radiotherapy (WBRT) (*n* = 6), surgery followed by adjuvant radiotherapy (*n* = 11), posterior fossa radiotherapy (PFRT) (*n* = 2), and palliative care (*n* = 2) (Table 1). The median follow-up since the diagnosis of brain metastases (BrM) was 7 months (range: 0.4–55.4 months). The median overall survival (OS) after BrM diagnosis was 9.4 months (95% CI: 4.8–14.8 months).

Univariate analysis revealed that the type of BrM treatment, age, number of BrM at diagnosis, ECOG status, and presence of extracranial disease were not significantly associated with improved OS. When OS was further stratified at 6, 12, and 18 months, analyzing single vs. multiple BrMs and CSPC vs. CRPC, no significant associations were found (Supplementary Tables S1 and S2). However, the total GPA score showed a trend toward significance (*P* = .07). Multivariable analysis further supported this finding, showing an upward trend in OS with higher GPA scores (*P* = .07) (Table 2).

**Table 2.** Univariate and Multivariable Hazard Ratios (HR) for Overall Survival from Brain Metastasis Diagnosis Please note that “Unadjusted HR” and “*p*” indicate univariate results of estimated HR and *P* value, and “Adjusted HR” and ‘*p* (adj)’ indicate multivariable results. BrM: brain metastasis; ECOG: Eastern Cooperative Oncology Group; GPA: Graded Prognostic Assessment; PFRT: posterior fossa radiotherapy; RT: radiotherapy; SRS: stereotactic radiosurgery; WBRT: whole brain radiotherapy

	Unadjusted HR (95%CI)	*p*	Adjusted HR (95%CI)	*p* (adj)
**BrM treatment**		0.8394		0.3610
SRS/WBRT/PFRT	Reference		Reference	
Surgery + RT	0.91 (0.36, 2.31)		1.73 (0.53, 5.60)	
**Age**	1.02 (0.97, 1.07)	0.5376		
**Number of BrM at diagnosis**	1.05 (0.90, 1.22)	0.5433		
**ECOG**		**0.4081**		0.5673
0	Reference		Reference	
1	0.55 (0.15, 2.06)	0.3782	0.29 (0.05, 1.63)	0.1595
2	0.70 (0.15, 3.33)	0.6577	0.26 (0.03, 2.11)	0.2089
3	1.70 (0.33, 8.69)	0.5254	0.34 (0.04, 3.26)	0.3476
**Total GPA score**	0.49 (0.23, 1.05)	**0.0674**	0.37 (0.13, 1.09)	**0.0711**
**Extracranial disease**		0.8586		
Present	Reference			
Negative	0.83 (0.11, 6.30)			

**Figure 4.**
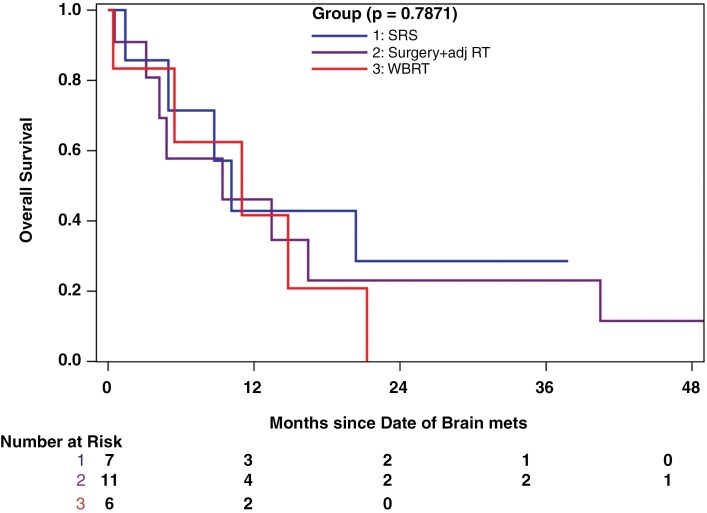
Overall Survival Distribution by Brain Metastasis Treatment Modality. RT: radiotherapy; SRS: stereotactic radiosurgery; WBRT: whole brain radiotherapy

Treatment modalities—surgery plus adjuvant radiation (9.4 months), SRS (10.1 months), or WBRT (11.0 months)—showed no significant difference in median survival (*P* = .79) (Figure 4). Although the median survival for patients with a single BrM (10.1 months) was higher than for those with multiple BrM (2.8 months), this difference did not reach statistical significance (*P* = .79). Likewise, median survival did not significantly differ between patients with castrate-sensitive prostate cancer (CSPC) (5.0 months) and those with castrate-resistant prostate cancer (CRPC) (10.1 months) (*P* = .69).

## Discussion

This 16-year retrospective review reveals a 0.7% incidence of BrM from PC, aligning with findings from similar retrospective series and underscoring their rarity within this patient demographic.^5–10^ However, the incidence rate might be influenced by the study’s institutional scope rather than a broader population-based approach. In addition, the patient sample in this study was only derived from a prospective database of patients referred to the BrM clinic by various subspecialty clinics. This may exclude some patients not referred by the genitourinary clinic and were managed independently instead, potentially introducing a selection bias. Unlike the MD Anderson Cancer Center (MDACC) study,^7^ which incorporated autopsy reports—identifying 60% of PC BrM cases post-mortem—our review’s exclusion of such reports likely underestimates asymptomatic BrM instances only discoverable at autopsy. The use of magnetic resonance imaging (MRI) for screening could potentially identify smaller, asymptomatic BrM, suggesting a possibly higher incidence rate than currently reported. However, the frequency with which MRIs are utilized for screening asymptomatic PC patients is not known in our study.

While some studies on prostate cancer with brain metastases excluded cases with dural-based disease, our review included both dural-based and purely parenchymal metastases. We identified a predominance of purely dural-based brain metastases from prostate cancer, accounting for 54% of the cases. In our study, skull-based metastases and dural-based metastases were distinguished using brain MRI, with the former showing involvement of the skull cortex and the latter limited to the pachymeningeal dura without bony involvement. Leptomeningeal metastases, on the other hand, were identified by enhancement or sugar coating pattern, typically observed in the cortical sulci or cerebellar folia.^11^ This contrasts with findings from a study conducted in Turkey,^5^ which reported a 20% incidence of pure dural-based disease, and the MDACC study, which reported an incidence of 21%.^7^ The overall incidence of BrM in the Turkish study was 2.9%, whereas the MDACC study reported an incidence of 0.8%. In our study, the overall incidence of pure-dural-based BrM is 0.38% (15 per 3,936 patients), while the incidence of pure intraparenchymal BrM is 0.30% (12 per 3,936 patients).

Understanding the pathways through which PC metastasizes—either hematogenously via the vena cava or retrogradely through the valveless Batson’s plexus—sheds light on its potential to reach distant sites, including the brain.^12,13^ Retrospective studies have shown certain PC histologies, such as small cell carcinoma (SCC) and neuroendocrine subtypes, are more prone to BrM, mirroring the metastatic behavior of SCC from lung cancer.^6,7,14,15^ In contrast, our study found only adenocarcinoma histology in all patients, with no instances of SCC or other rare subtypes. In an analysis of BrM from PC of purely adenocarcinoma origin by Bhambhvani et al., the proportion of BrM from PC is 0.86%, a rate that closely parallels the incidence found in our study.^10^

Consistent with prior reports, our study found that BrM from PC predominantly occurred in patients presenting with high-risk features such as elevated PSA levels at diagnosis, advanced Gleason scores, PNI, castrate resistance, and synchronous metastases in other organs.^5–7,10,16^ Furthermore, the median interval between PC diagnosis and BrM detection—47.8 months—parallels findings from other studies.^5,6,14,17,18^ This pattern underscores the potential need for targeted BrM screening strategies at a certain time point in patients with these high-risk features. The majority of patients do exhibit symptoms, however, typically presenting with at least one non-focal symptom, whereas seizures are uncommon. This highlights the complexity of BrM symptomatology in PC and further supports the rationale for regular monitoring in this patient population. The true incidence of brain metastases from prostate cancer is likely underestimated, as evidenced by the MDACC study, where 78 cases were diagnosed via autopsy compared to 53 identified through imaging. Improved survival rates in prostate cancer may contribute to a rising incidence of brain metastases. For instance, a study by Caffo et al. reported an increase in brain metastases from 0.8% to 2.8% following the introduction of docetaxel, with most cases occurring after patients developed castration-resistant disease.^17^ Currently, no published guidelines exist on how to screen for brain metastases in high-risk prostate cancer patients. However, the increasing use of prostate-specific membrane antigen positron emission tomography (PSMA-PET) has shown promise in detecting incidental brain lesions.^19–22^ Equivocal findings on PSMA-PET could then be further evaluated with MRI for detailed characterization. This approach seems reasonable for monitoring high-risk patients.

The median survival in our study was 9.4 months, which is higher than most institutional retrospective analyses reporting 2.8–5.5 months.^5–10^ Population-based database studies have reported longer median survivals of 10–12 months.^23,24^ Specifically, our study identified a median survival of 10.1 months in BrM patients treated with SRS, comparable to the MDACC study’s 9 months.^7^ Another study involving patients exclusively treated with Gamma Knife SRS reported a median survival of 13 months.^14^ Notably, in our study, the SRS group exhibited a relatively higher 6-month survival rate compared to other groups (SRS: 71.4%; surgery and adjuvant RT: 57.7%; WBRT: 62.5%), although this advantage did not extend beyond 12 months (Supplementary Table S3). Patients in our study treated with WBRT had a median survival of 11 months, higher than the 2–7 months reported in other retrospective studies.^5,8,25,26^ Nonetheless, these comparisons are limited by our study’s small sample size and the variations in the time periods during which patients were treated, potentially affecting the availability of diagnostic and treatment modalities. At our center, the decision to administer WBRT was guided by the number of brain metastases and the patient’s performance status. Regarding stereotactic treatment, fractionated SRS was introduced in 2017, particularly for large brain metastases, whereas single-fraction SRS was the standard practice prior to 2017. Detailed information on dose, volume, and fractionation has been previously described.^27^

Our study did not identify any patient or treatment-related factors that significantly correlated with OS. However, a higher GPA score showed a trend toward improved OS. The GPA is a well-established prognostic index for patients with brain metastases. Over time, the GPA has been refined with diagnosis-specific prognostic indices for breast cancer, small cell and non-small cell lung cancer, gastrointestinal cancer, melanoma, and renal cell carcinoma.^28^ The rarity of BrM from PC may make it challenging to identify prognostic factors specific to this disease. Nonetheless, the trend toward improved OS with a higher generic GPA score is noteworthy. Future studies in larger patient groups should investigate disease-specific factors that could potentially predict outcomes for this group of patients.

This study has several limitations. Firstly, it is a retrospective analysis conducted at a single tertiary center, which may introduce selection bias. PC patients with BrM who were not referred to the BrM Clinic for management were not included, potentially skewing the data. In addition, autopsy reports were not incorporated. The low incidence of BrM from PC resulted in a small sample size, limiting the ability to draw definitive conclusions regarding predictive factors for OS. To address these limitations, future research should include longitudinal studies with long-term follow-up to help identify late-emerging prognostic factors and trends. Investigating genetic and molecular markers may uncover novel prognostic factors specific to PC patients. Ultimately, future studies should aim to develop a disease-specific GPA for PC patients with BrM to improve prognostic accuracy and guide treatment decisions.

## Conclusion

BrM from PC are rare, predominantly occurring in patients with advanced, castrate-resistant stages, and are often accompanied by other metastases. This analysis aids in understanding the disease trajectory and informing treatment discussions. Despite the rarity and the challenges in identifying specific prognostic factors, the study highlights the potential role of the GPA as a prognosis tool to estimate OS. Future research should focus on longitudinal studies with long-term follow-up to identify late-emerging prognostic factors.

## Supplementary Material

vdaf063_suppl_Supplementary_Table_S1

vdaf063_suppl_Supplementary_Table_S2

vdaf063_suppl_Supplementary_Table_S3

## Data Availability

De-identified data may be available upon reasonable request to the corresponding author. Please email Dr. Enrique Gutierrez at Enrique.Gutierrez@uhn.ca
