# Supplementary material for: Survival outcomes and clinical characteristics of brain metastases from prostate cancer: A single-center analysis
Source: Neurooncol Adv. 2025 Mar 22;7(1):vdaf063. doi: 10.1093/noajnl/vdaf063 (PMC12082812; doi:10.1093/noajnl/vdaf063)
Supplement: vdaf063_suppl_Supplementary_Table_S1 [file vdaf063_suppl_supplementary_table_s1.docx]

**Supplementary Table 1.** Overall survival rates at 6, 12, and 18 months from with 95% confidence intervals for different treatment groups

| **Group** | **Month** | **Survival** | **95% CI** | |
| --- | --- | --- | --- | --- |
|  |  |  | **Lower** | **Upper** |
| Overall | 6 | 0.573 | 0.361 | 0.738 |
|  | 12 | 0.409 | 0.220 | 0.591 |
|  | 18 | 0.246 | 0.100 | 0.424 |
| SRS | 6 | 0.714 | 0.258 | 0.920 |
|  | 12 | 0.429 | 0.098 | 0.734 |
|  | 18 | 0.429 | 0.098 | 0.734 |
| Surgery + adjuvant radiotherapy | 6 | 0.577 | 0.221 | 0.819 |
|  | 12 | 0.462 | 0.145 | 0.734 |
|  | 18 | 0.231 | 0.035 | 0.526 |
| WBRT | 6 | 0.625 | 0.142 | 0.893 |
|  | 12 | 0.417 | 0.056 | 0.767 |
|  | 18 | 0.208 | 0.009 | 0.595 |
