# Supplementary material for: Survival outcomes and clinical characteristics of brain metastases from prostate cancer: A single-center analysis
Source: Neurooncol Adv. 2025 Mar 22;7(1):vdaf063. doi: 10.1093/noajnl/vdaf063 (PMC12082812; doi:10.1093/noajnl/vdaf063)
Supplement: vdaf063_suppl_Supplementary_Table_S2 [file vdaf063_suppl_supplementary_table_s2.docx]

**Supplementary Table 2.** Overall survival rates at 6, 12, and 18 months from with 95% confidence intervals for single versus multiple brain metastases

| **Number of brain metastases** | **Month** | **Survival** | **95% CI** | |
| --- | --- | --- | --- | --- |
|  |  |  | **Lower** | **Upper** |
| Multiple | 6 | 0.696 | 0.378 | 0.874 |
|  | 12 | 0.387 | 0.142 | 0.630 |
|  | 18 | 0.155 | 0.025 | 0.389 |
| Single | 6 | 0.445 | 0.172 | 0.690 |
|  | 12 | 0.445 | 0.172 | 0.690 |
|  | 18 | 0.356 | 0.114 | 0.612 |
