# Supplementary material for: Survival outcomes and clinical characteristics of brain metastases from prostate cancer: A single-center analysis
Source: Neurooncol Adv. 2025 Mar 22;7(1):vdaf063. doi: 10.1093/noajnl/vdaf063 (PMC12082812; doi:10.1093/noajnl/vdaf063)
Supplement: vdaf063_suppl_Supplementary_Table_S3 [file vdaf063_suppl_supplementary_table_s3.docx]

**Supplementary Table 3.** Overall survival rates at 6, 12, and 18 months from with 95% confidence intervals for castrate-sensitive versus castrate-resistant prostate cancer

| **CSPC vs CRPC** | **Month** | **Survival** | **95% CI** | |
| --- | --- | --- | --- | --- |
|  |  |  | **Lower** | **Upper** |
| CSPC | 6 | 0.417 | 0.056 | 0.767 |
|  | 12 | 0.417 | 0.056 | 0.767 |
|  | 18 | 0.417 | 0.056 | 0.767 |
| CRPC | 6 | 0.612 | 0.370 | 0.784 |
|  | 12 | 0.408 | 0.198 | 0.608 |
|  | 18 | 0.204 | 0.064 | 0.399 |
